# Optimization Method for Secrecy Capacity of UAV Relaying Based on Dynamic Adjustment of Power Allocation Factor

**DOI:** 10.3390/s26020592

**Published:** 2026-01-15

**Authors:** Yunqi Hao, Youyang Xiang, Qilong Du, Xianglu Li, Chen Ding, Dong Hou, Jie Tian

**Affiliations:** 1 Institute of Electronic Engineering, China Academy of Engineering Physics, Mianyang 621999, China; haoyunqi23@gscaep.ac.cn (Y.H.);; 2 95875 Unit, Jiuquan 735000, China; 3 The School of Automation Engineering, University of Electronic Science and Technology of China, Chengdu 611731, China

**Keywords:** physical layer security, UAV, artificial noise, power allocation factor, secrecy capacity

## Abstract

The broadcast nature of wireless channels introduces significant security vulnerabilities in information transmission, particularly when the eavesdropper is close to the legitimate destination. In such scenarios, the eavesdropping channel often exhibits high spatial correlation with, or even superior quality to, the legitimate channel. This makes it challenging for traditional power optimization methods to effectively suppress the eavesdropping rate. To address this challenge, this paper proposes an optimization method for the secrecy capacity of unmanned aerial vehicle (UAV) relaying based on the dynamic adjustment of the power allocation factor. By injecting artificial noise (AN) during signal forwarding and combining it with real-time channel state information, the power allocation factor can be dynamically adjusted to achieve precise jamming of the eavesdropping link. We consider a four-node communication model consisting of a source, a UAV, a legitimate destination, and a passive eavesdropper, and formulate a joint optimization problem to maximize the secrecy rate. Due to the non-convexity of the original problem, we introduce relaxation variables and apply successive convex approximation (SCA) to reformulate it into an equivalent convex optimization problem. An analytical solution for the power allocation factor is derived using the water-filling (WF) algorithm. Furthermore, an alternating iterative optimization algorithm with AN assistance is proposed to achieve global optimization of the system parameters. Simulation results demonstrate that, compared to traditional power optimization schemes, the proposed algorithm substantially suppresses the eavesdropping channel capacity while enhancing transmission efficiency, thereby significantly improving both secrecy performance and overall communication reliability.

## 1. Introduction

In recent years, UAVs have revolutionized wireless communication paradigms by capitalizing on their exceptional mobility, flexible deployment, and cost-effectiveness. They facilitate the rapid establishment of temporary communication links, significantly extend network range, and enhance signal transmission quality [1], thereby serving as pivotal aerial relays in diverse application scenarios such as informatized warfare, disaster response, remote area connectivity, and heterogeneous networking [2,3]. Within integrated space-air-ground networks, UAVs effectively bridge coverage gaps by provisioning reliable line-of-sight (LoS) links, thereby bolstering overall network connectivity and resilience [4,5]. However, the inherent broadcast nature of wireless channels makes transmitted signals susceptible to interception by unauthorized parties, leading to potential information leakage risks, which presents security challenges for UAV communication systems [6].

Traditional secure communication systems predominantly rely on cryptographic techniques (e.g., asymmetric encryption, authentication, and access control) to safeguard information integrity and confidentiality [7,8,9]. While these methods effectively ensure security by increasing computational complexity, they often incur substantial computational overhead and require complex key management protocols [10]. Furthermore, the rapid advancement of quantum computing has begun to erode the computational complexity assumptions underlying many classical cryptographic algorithms, thereby elevating the risk of their compromise [11,12,13].

Concurrently, the open nature of wireless channels in UAV communication systems has spurred interest in physical layer security (PLS) techniques [14,15]. PLS enhances security by exploiting inherent channel characteristics—such as fading, noise, and interference—to actively degrade the signal reception conditions at potential eavesdroppers [16]. Compared to traditional cryptographic approaches, PLS offers advantages in lower computational complexity and greater adaptability to dynamic environments. However, existing PLS frameworks face challenges in achieving optimal performance in complex and dynamic scenarios, particularly when the eavesdropper is in close proximity to the legitimate destination. Under such conditions, the highly correlated CSI between the legitimate and eavesdropping channels severely limits the effectiveness of PLS mechanisms [17,18,19].

The application of UAVs for information transmission was pioneered in [20], where trajectory design and power allocation were optimized via convex optimization methods, laying the groundwork for subsequent research in secure communications. Ref. [21] extended this framework by introducing the presence of an eavesdropper and proposing an iterative difference-of-convex (DC) programming algorithm to maximize the secrecy capacity through adaptive power allocation based on channel disparity. However, this single power optimization algorithm requires a certain degree of separation between the legitimate and eavesdropping channels, implying that information transmission is effective only when the legitimate channel is superior to the eavesdropping channel. This inherent constraint limits its applicability in scenarios where channel conditions are highly correlated or adversarial. To tackle this issue, refs. [22,23,24] jointly optimized UAV trajectory and power under eavesdropper position uncertainty, ensuring worst-case secrecy rate guarantees by enlarging legitimate-eavesdropper channel differences via trajectory design. Refs. [25,26,27] propose to maximize the secrecy capacity by optimizing the UAV’s hovering position (3D coordinates), where the optimal hovering location is determined through analyzing channel gain disparities. Nevertheless, when the eavesdropper is in close proximity to the legitimate destination, such location-trajectory-power methods can’t effectively suppress the eavesdropping rate.

To counteract eavesdropping threats, the study in [28] proposed deploying a UAV as a friendly jammer, optimizing its placement strategy to minimize the eavesdropper’s interception probability without requiring prior knowledge of the eavesdropper’s location. In [29], the secure remote control (RC) problem in multi-UAV systems was investigated, introducing cooperative jamming (CJ) among UAVs to safeguard control data transmitted between UAVs and ground base stations. Similarly, ref. [30] employed cooperative jamming with multi-node AN to degrade the eavesdropper’s channel conditions effectively. Further advancing this field, ref. [31] integrated AN with three-dimensional trajectory optimization for multi-user and multi-eavesdropper scenarios, achieving significant secrecy gains in dense urban environments. Ref. [32] overcame the limitations of traditional power allocation by jointly optimizing the energy allocation between the signal and the AN, thereby reducing the eavesdropper’s interception probability while maintaining robustness across diverse scenarios.

To address the challenge of traditional power optimization methods failing to effectively suppress eavesdropping capacity when eavesdroppers are in close proximity to legitimate destinations, this paper proposes a physical-layer security enhancement technique for UAV relay systems assisted by AN. The proposed technique adaptively controls interference on eavesdropping channels by adjusting the signal-to-AN power allocation factor based on the UAV’s dynamic position and real-time CSI. Considering the information causality constraint, which ensures that the UAV relay can only forward information received from the source, we jointly optimize the source transmission power and the power allocation factor to improve both information transmission efficiency and system security. An alternating iterative algorithm is designed to adjust these parameters, maximizing the secrecy capacity while satisfying all constraints. The secrecy capacity maximization problem is inherently non-convex and computationally intractable. The main contributions of this paper are as follows: (1) A dynamic and precise jamming control mechanism for UAV is proposed, targeting severe “proximity eavesdropping” scenarios. This paper overcomes the inherent reliance on channel disparity in traditional UAV secure communication research and effectively addresses the challenge of traditional methods failing when the eavesdropper is in close proximity to the legitimate destination. (2) By integrating slack variables with the SCA technique, analytical solutions for the optimal source power allocation and UAV jamming power allocation factors are derived. This avoids the high computational complexity of traditional iterative algorithms, reducing the complexity to $O\left(N^{3}\right)$. (3) The algorithm achieves a synergistic optimization of security performance and communication efficiency. While maintaining secrecy capacity without degradation, the proposed scheme intelligently schedules resources to reduce eavesdropping capacity by approximately 79% and improve communication efficiency by about 9%, thereby breaking the traditional trade-off between security and efficiency.

The remainder of the paper is organized as follows: Section 2 presents the system model, Section 3 details the system design and optimization approach, Section 4 discusses the simulation results, and Section 5 provides the conclusion.

## 2. System Model

**Figure 1:**
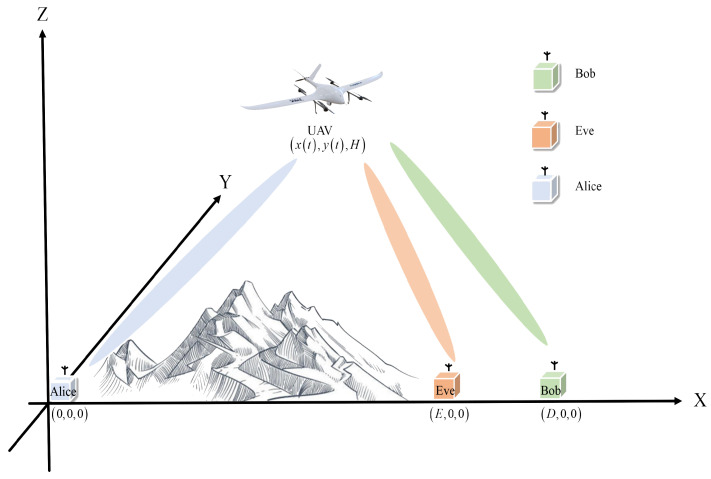
The illustration of the UAV uplink and downlink.

Consider a wireless communication system comprising a source node (Alice), a legitimate destination (Bob), a UAV relay, and an eavesdropper (Eve). Due to terrain obstructions, the line-of-sight (LoS) link between Alice and Bob is blocked, and the channel gain is negligible due to severe shadow fading. To facilitate reliable communication, a UAV is deployed as a mobile relay node to dynamically forward data between Alice and Bob. To mitigate the risk of sensitive information leakage, we assume that Eve has compromised a node in close proximity to Bob, necessitating robust countermeasures to prevent data interception and ensure secure communication. For clarity, as shown in Figure 1, we adopt a Cartesian coordinate system to represent positions. Alice, Eve, and Bob are positioned at coordinates (0,0,0),  (E,0,0), and  (D,0,0), respectively. The UAV is initially located directly above Alice at a fixed altitude (H) and moves toward Bob’s position. The flight duration mobile(T) is discretized into (N) equally spaced time slots, and the UAV’s position coordinates can be expressed as $\left(x\left[n\right],y\left[n\right],H\right)$.

During the flight, the UAV continuously receives information from Alice and forwards it to Bob under the condition of secure transmission.  To ensure communication security, the UAV injects AN while relaying information, thereby interfering with the eavesdropping channel. To enable simultaneous information reception and forwarding by the UAV, mitigate multipath interference and adjacent-channel interference, and enhance the anti-jamming capability of both uplink and downlink, we assume the UAV operates in   Frequency Division Duplexing (FDD) mode  with an infinite data buffer and allocates equal bandwidth to Alice and Bob. The channel gains for the Alice–UAV, UAV–Eve, and UAV–Bob links are denoted as $h_{sr}\left[n\right]$, $h_{re}\left[n\right]$ and $h_{rd}\left[n\right]$; it follows the Free Space Path Loss (FSPL) model: 

(1) $$h_{ij}\left[n\right]=\beta_{0}d_{ij}^{-2}\left[n\right],ij\in \left\{sr,re,rd\right\},n=1,2,\ldots ,N,$$

The link distance of Alice–UAV is denoted as $d_{sr}\left[n\right]=\sqrt{x^{2}\left[n\right]+y^{2}\left[n\right]+H^{2}}$; the link distance of UAV–Eve is denoted as $d_{re}\left[n\right]=\sqrt{{\left(E-x\left[n\right]\right)}^{2}+y^{2}\left[n\right]+H^{2}}$; and the link distance of UAV–Bob is denoted as $d_{rd}\left[n\right]=\sqrt{{\left(D-x\left[n\right]\right)}^{2}+y^{2}\left[n\right]+H^{2}}$. $\beta_{0}$ denotes channel power gain at the reference distance $d_{0}=1$ m.

Let γ0=β0β0σ2σ2 denote the signal-to-noise ratio (SNR), where $\sigma^{2}$ is the noise power. Without AN injection, the SNR of each link is expressed as follows:

(2) $$\gamma_{ij}\left[n\right]=\frac{\beta_{0}d_{ij}^{-2}\left[n\right]}{\sigma^{2}},ij\in \left\{sr,rd,re\right\},n=1,2,\ldots ,N.$$

We assume the power allocation factor $w\left[n\right]$ to control the transmission power of the signal and AN. Assuming the UAV’s total transmit power in a unit time slot is *P*, the AN is shared between the UAV and Bob. Since Bob has prior knowledge of the AN, the AN’s interference does not degrade Bob’s reception performance.  After being interfered with by AN, the signal-to-interference-plus-noise ratio (SINR) of the UAV–Eve link is expressed as follows:

(3a) $$\begin{array}{cc} SINR_{re}\left[n\right] & =\frac{h_{re}\left[n\right]}{\left(1-w\left[n\right]\right)P\cdot h_{re}\left[n\right]+\sigma^{2}} \end{array}$$

(3b) $$\begin{array}{cc} \; & \;\;\;\;\;\;\;\;=\frac{\gamma_{re}\left[n\right]}{\left(1-w\left[n\right]\right)P\cdot \gamma_{re}\left[n\right]+1},n=1,2,\ldots N, \end{array}$$

where $w\left[n\right]$ satisfies the constraint $0\leq w\left[n\right]\leq 1$; when $w\left[n\right]=1$, it indicates that no AN is added, and all the power *P* is used for information transmission. When $w\left[n\right]=0$, it means no information is transmitted, and all the power *P* is used for sending AN. To optimize the system’s energy efficiency, a power-shutdown strategy can be implemented during the $w\left[n\right]=0$ period, in which the transmission power is set to zero during that time.

According to the Shannon formula, the theoretical information transmission rates of the UAV, Eve, and Bob are given by the following:

(4) $$R_{s}\left(\left\{p_{s}\left[n\right]\right\}\right)=\log_{2}\left(1+p_{s}\left[n\right]\gamma_{sr}\left[n\right]\right),n=1,2,\ldots ,N,$$

(5) $$R_{d}\left(\left\{w\left[n\right]\right\}\right)=\log_{2}\left(1+w\left[n\right]P\cdot \gamma_{rd}\left[n\right]\right),n=1,2,\ldots ,N,$$

(6) $$R_{e}\left(\left\{w\left[n\right]\right\}\right)=\log_{2}\left(1+w\left[n\right]P\cdot SINR_{re}\left[n\right]\right),n=1,2,\ldots ,N.$$

## 3. Enhance Security by Designing Alternating Iterative Algorithms

To ensure the feasibility of the optimization problem, it is necessary to introduce information-causal constraints, power limitations, and other conditions to ensure that the optimal solution for the system parameters lies within the feasible domain. Based on the dynamic changes in CSI and the three-dimensional spatial coordinates of the UAV, the power allocation factor is adjusted to control the power ratio between the UAV transmission signals and AN, thereby achieving precise interference suppression of eavesdropping channels and significantly improving physical-layer security performance. This section has conducted in-depth analysis and optimization with the goal of maximizing confidentiality capacity.

### 3.1. Problem Formulation

To ensure the rationality of the transmission power and that the total transmission power of Alice in each time slot does not exceed the preset average power limit, there is a power constraint:

(7) $$\frac{1}{N}\sum_{n=1}^{N-1}p_{s}\left[n\right]\leq {\bar{P}}_{s},$$

Assuming Eve and Bob have the same channel conditions, define the difference between $R_{d}\left(\left\{w\left[n\right]\right\}\right)$ and $R_{e}\left(\left\{w\left[n\right]\right\}\right)$ as the secrecy capacity within a unit time slot. The total secrecy capacity of the system is as follows:

(8) $$C_{s}^{*}=\sum_{n=2}^{N}R_{d}\left(\left\{w\left[n\right]\right\}\right)-R_{e}\left(\left\{w\left[n\right]\right\}\right).$$

Moreover, to ensure that the UAV can only forward information received from Alice, there is an information causality constraint:

(9) $$\sum_{i=2}^{n}R_{d}\left[i\right]\leq \sum_{i=1}^{n-1}R_{s}\left[i\right],n=2,\cdots ,N,$$

where $R_{s}\left[N\right]=0,R_{d}\left[1\right]=0.$

In summary, the secure transmission problem of the proposed UAV relay system can be formulated as a constrained secrecy capacity maximization (SCM) problem:  

(10a) Cs*=maxpsnn=1N−1,wnn=2N∑n=2NRdwn−Rewn

(10b) $$\begin{array}{cc} s.t. & \sum_{i=2}^{n}R_{d}\left(\left\{w\left[n\right]\right\}\right)\leq \sum_{i=1}^{n-1}R_{s}\left(\left\{p_{s}\left[n\right]\right\}\right),n=2,\ldots ,N, \end{array}$$

(10c) $$\begin{array}{cc} \; & 0\leq w\left[n\right]\leq 1,n=2,\ldots ,N, \end{array}$$

(10d) $$\begin{array}{cc} \; & \frac{1}{N}\sum_{i=1}^{N-1}p_{s}\left[n\right]\leq {\bar{P}}_{s},n=1,2,\ldots ,N-1, \end{array}$$

(10e) $$\begin{array}{cc} \; & p_{s}\left[n\right]\geq 0,n=1,2,\ldots ,N-1, \end{array}$$

**Figure 2:**
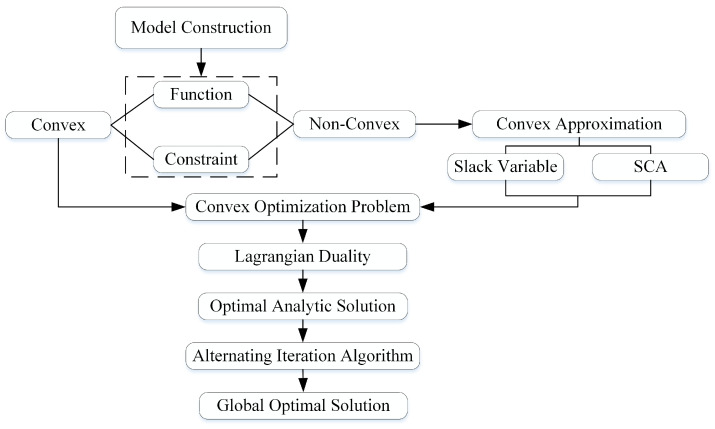
Convex Optimization Framework.

This paper formulates an optimization problem that contains both a non-convex objective function and non-convex constraints. The specific process is illustrated in Figure 2. First, to handle the non-convex components, a convex approximation approach is adopted: by introducing slack variables and employing the SCA technique, the original problem is transformed into a convex optimization problem. Subsequently, the Lagrangian dual method is applied to solve the convex problem, yielding an optimal analytical solution. Finally, an alternating iterative algorithm is used to iteratively optimize the variables, which ultimately converges to the global optimum.

### 3.2. Convex Approximation to the SCM Problem

The optimization problem is non-convex due to the (10a) and (10b), since non-convex problems may contain multiple local optima, direct solving methods are prone to getting trapped in local optimality, leading to unstable algorithm performance. Therefore, in this section, we first equivalently reformulate the original problem into a convex approximation.

For the non-convex constraint (10b), an equivalent secrecy capacity maximization problem is derived by introducing relaxation variables $R_{d}\left[n\right]$:

(11a) Cs*=maxpsnn=1N−1,wnn=1N−1∑n=2NRdn−Rewn

(11b) $$\begin{array}{cc} s.t. & \sum_{i=2}^{n}R_{d}\left[n\right]\leq \sum_{i=1}^{n-1}R_{s}\left(\left\{p_{s}\left[n\right]\right\}\right),n=2,\ldots ,N, \end{array}$$

(11c) $$\begin{array}{cc} \; & R_{d}\left[n\right]\leq R_{d}\left(\left\{w\left[n\right]\right\}\right),n=2,\ldots ,N, \end{array}$$

(11d) $$\begin{array}{cc} \; & (10c)-(10e)\;\mathrm{satisfied}. \end{array}$$

To further reduce the complexity of (11a), the SCA method is employed to transform problem (11a) into a sequence of convex subproblems. In essence, this involves approximating the objective function $R_{e}\left(\left\{w\left[n\right]\right\}\right)$ at a feasible point $w_{0}\left[n\right]$ using its first-order Taylor expansion for the SCM problem:

(12) Cs*=maxpsnn=1N−1,wnn=1N−1∑n=2NRdn−∑n=2NRew0n−∑n=2Nγrenwn−w0n1−w0nγren+11PPln2s.t.(11b)−(11d)satisfied.

Note that (12) is a convex problem, which can be solved by standard convex optimization techniques. Compared to the original problem, it has a tighter convergence property, manifested as follows:

1. The feasible domain shrinks, and by introducing relaxation variables, the solution space of the original problem is constrained to a more compact convex set.
2. The objective function is simpler and easier to solve.
3. The convergence speed is improved, and the equivalent convex problem can quickly converge to the global optimal solution.

### 3.3. Secrecy Capacity Optimization Through Alternating Iterative Algorithm Design

To reduce the complexity of the feasible domain for equivalent convex optimization problems, this paper uses Lagrange dual functions to incorporate causal constraints (11b) into the objective function, thereby transforming the constraints into penalty terms of the objective function and avoiding dealing with complex constraints.

Introducing dual variables ${\left\{\lambda_{n}\right\}}_{n=2}^{N}$ and using Lagrange dual functions to solve (12):

(13) Lpsn,wn,Rdn,λn=∑n=2NRdn−∑n=2NRew0n−∑n=2Nγrenwn−w0n1−w0nγren+11PPln2+∑n=2Nλn∑i=1n−1log1+psiγsri−∑i=2nRdi=∑n=2NνnRdn−∑n=2NRew0n−∑n=2Nγrenwn−w0nγren+11PPln2+∑n=1N−1βnlog1+psnγsrns.t.(11b)−(11d)satisfied.

where $\beta_{n}=\sum_{i=n+1}^{N}\lambda_{i},n=1,\ldots ,N-1,$$\nu_{n}=1-\sum_{i=n}^{N}\lambda_{i},n=2,\ldots ,N.$

Based on the variables $p_{s}\left[n\right]$,$w\left[n\right]$ and ${\left\{\lambda_{n}\right\}}_{n=2}^{N}$, the objective problem can be divided into two sub-problems:

(14) $$\begin{array}{cc} \max_{\left\{p_{s}\left[n\right]\right\}} & \sum_{n=1}^{N-1}\beta_{n}\log_{2}\left(1+p_{s}\left[n\right]\gamma_{sr}\left[n\right]\right)\\ s.t. & \frac{1}{N}\sum_{n=1}^{N-1}p_{s}\left[n\right]\leq {\bar{P}}_{s}\\ \; & p_{s}\left[n\right]\geq 0,n=1,2,\ldots ,N-1. \end{array}$$

(15) maxwn∑n=2NνnRdn−∑n=2Nγrenwn−w0n1−w0nγren+11PPln2s.t.Rdn≤Rdwn,n=2,…,N,0≤wn≤1,n=2,…,N,

where $\lambda_{n}\geq 0,\forall n$. To ensure that the optimal solution of (15) is bounded, it is necessary to ensure that $v_{n}\geq 0,\forall n$, i.e., $\sum_{n=2}^{N}\lambda_{n}\leq 1.$

Equation (12) satisfies the Slater condition, so the optimal solution of the original problem is equal to the optimal solution of the dual problem. The proof is detailed as follows:

**Proof.**  Assume $p_{s}\left[n\right]=\varepsilon_{s},\forall n$, where $0<\varepsilon_{s}<\min\left\{{\bar{P}}_{s},\frac{N}{N-1}{\bar{P}}_{s}\right\}$. Simultaneously, let $w_{1}\left[n\right]=w_{0}\left[n\right]+\delta_{w},\forall n$, where $0<\delta_{w}<\min\left\{1-w_{0}\left[n\right],w_{0}\left[n\right]\right\}$. Under this parameter construction, the power constraints are strictly satisfied:

(16) $$\begin{array}{c} \begin{array}{c} \frac{1}{N}\sum_{n=1}^{N-1}p_{s}\left[n\right]=\frac{\left(N-1\right)\varepsilon_{s}}{N}<{\bar{P}}_{s}\\ p_{s}\left[n\right]=\varepsilon_{s}>0,\;0<w\left[n\right]<1. \end{array} \end{array}$$

Since $R_{s}\left[n\right]=\log_{2}\left(1+\varepsilon_{s}\gamma_{sr}\left[n\right]\right)>0$ and $R_{d}\left[n\right]=\log_{2}\left(1+w_{1}\left[n\right]P\cdot \gamma_{rd}\left[n\right]\right)>0$, and both are continuous, strictly increasing functions of their arguments, we can choose sufficiently small $\varepsilon_{s}$ and $\delta_{w}$ such that

(17) $$\varepsilon_{s}\gamma_{sr}\left[i\right]\ll 1,\;w\left[i\right]P\cdot \gamma_{rd}\left[i\right]\ll 1.$$

Using the approximation log21+x≈xxln2ln2 for small *x*, we have the following:

(18) $$\begin{array}{c} \begin{array}{c} \sum_{i=1}^{n-1}R_{s}\left[i\right]\approx \frac{\varepsilon_{s}}{\ln2}\sum_{i=1}^{n-1}\gamma_{sr}\left[i\right],\;\sum_{i=2}^{n}R_{d}\left[i\right]\approx \frac{P\delta_{w}}{\ln2}\sum_{i=1}^{n-1}\gamma_{rd}\left[i\right]. \end{array} \end{array}$$

By choosing $\varepsilon_{s}$ sufficiently larger than $\delta_{w}$(e.g., $\varepsilon_{s}=\kappa \delta_{w}$ with $\kappa >\frac{P\sum_{i=2}^{n}\gamma_{rd}\left[i\right]}{\sum_{i=1}^{n-1}\gamma_{sr}\left[i\right]}$), the strict inequality holds.

The rate constraint (11c) may choose $R_{d}\left[n\right]$ appropriately close to but strictly less than $R_{d}\left(\left\{w\left[n\right]\right\}\right)$. Since all constraints can be satisfied with strict inequalities, Slater’s condition holds.    □

(1) For any given $\lambda_{n}$, using the Lagrange multiplier method to solve (14) and (15), we obtain the following:

(19) $$\begin{array}{cc} L\left(p_{s}\left[n\right],\eta ,\alpha_{n}\right)= & -\sum_{n=1}^{N-1}\beta_{n}\log_{2}\left(1+p_{s}\left[n\right]\gamma_{sr}\left[n\right]\right)+\eta \left(\frac{1}{N}\sum_{n=1}^{N-1}p_{s}\left[n\right]-{\bar{P}}_{s}\right)\\ \; & -\sum_{n=1}^{N-1}\alpha_{n}p_{s}\left[n\right] \end{array}$$

(20) Lwn,ϖn,ϑn,θn,=∑n=2Nγrenwn−w0n1−w0nγren+11PPln2−∑n=2NνnRdn+ϖnRdn−Rdwn−∑n=2Nϑnwn+∑n=2Nθnwn−1

(21) $$g_{s}\left(\lambda_{n}\right)=\underset{p_{s}\left[n\right]}{\inf}L\left(p_{s}\left[n\right],\eta ,\alpha_{n}\right)$$

(22) $$g_{r}\left(\lambda_{n}\right)=\underset{w\left[n\right]}{\inf}L\left(w\left[n\right],\varpi_{n},\theta ,\vartheta_{n}\right)$$

Equation (21) satisfies the following KKT condition:

(23a) $$\begin{array}{c} \frac{-\beta_{n}\gamma_{sr}\left[n\right]}{\left(1+p_{s}\left[n\right]\gamma_{sr}\left[n\right]\right)\ln2}+\eta -\alpha_{n}=0, \end{array}$$

(23b) $$\begin{array}{c} \alpha_{n}\geq 0,p_{s}\left[n\right]\geq 0,\alpha_{n}p_{s}\left[n\right]=0, \end{array}$$

(23c) $$\begin{array}{c} \eta \geq 0,\eta \left(\frac{1}{N}\sum_{n=1}^{N-1}p_{s}\left[n\right]-{\bar{P}}_{s}\right)=0, \end{array}$$

where η is the parameter ensuring the equality   $\frac{1}{N}\sum_{i=1}^{N-1}p_{s}\left[n\right]={\bar{P}}_{s}$, while   ${\left\{\alpha_{n}\right\}}_{n=1}^{N-1}$ is Lagrange multiplier corresponding to $p_{s}\left[n\right]\geq 0$.

When $\eta <\left(\log_{2}e\right)\beta_{n}\gamma_{sr}\left[n\right]$, $\alpha_{n}=0$, from Equation (23a), we can obtain the following:

(24) $$p_{s}\left[n\right]=\frac{\beta_{n}}{\eta \ln2}-\frac{1}{\gamma_{sr}\left[n\right]}.$$

When $\eta \geq \left(\log_{2}e\right)\beta_{n}\gamma_{sr}\left[n\right],\alpha_{n}>0$, it follows that $p_{s}\left[n\right]=0$.

Equation (22) satisfies the following KKT condition:

(25a) γren1−w0nγren+11PPln2−νnP·γrdn1+wnP·γrd[n]ln2−ϑn+θn=0,

(25b) $$\begin{array}{c} \;\;\;\;\;\;\;\;\;\;\;\;\;\;\;\;\;\;v_{n}=\varpi_{n},\varpi_{n}\geq 0,\vartheta_{n}\geq 0,\theta_{n}\geq 0,\\ \vartheta_{n}w\left[n\right]=0,\theta_{n}\left(w\left[n\right]-1\right)=0, \end{array}$$

(25c) $$\begin{array}{c} \;\;\;\;\;\;\;\;\;\;\;\;\;\;\varpi_{n}\left(R_{d}\left[n\right]-R_{d}\left(\left\{w\left[n\right]\right\}\right)\right)=0,n=2,\cdots ,N, \end{array}$$

where $\varpi_{n}$ is the parameter ensuring the equality $R_{d}\left[n\right]=R_{d}\left(\left\{w\left[n\right]\right\}\right)$ holds, $\vartheta_{n}$ and   $\theta_{n}$ are Lagrange multipliers corresponding to $0\leq w\left[n\right]\leq 1$.

When $\nu_{n}\leq \frac{\gamma_{re}\left[n\right]}{\left(\left(1-w_{0}\left[n\right]\right)P\cdot \gamma_{re}\left[n\right]+1\right)\gamma_{rd}\left[n\right]}$ and $\theta_{n}=0,\vartheta_{n}\geq 0,$ it follows that $w\left[n\right]=0$.

When $\frac{\gamma_{re}\left[n\right]}{\left(\left(1-w_{0}\left[n\right]\right)P\cdot \gamma_{re}\left[n\right]+1\right)\gamma_{rd}\left[n\right]}<\nu_{n}<\frac{\gamma_{re}\left[n\right]\left(1+P\cdot \gamma_{rd}\left[n\right]\right)}{\left(\left(1-w_{0}\left[n\right]\right)P\cdot \gamma_{re}\left[n\right]+1\right)\gamma_{rd}\left[n\right]}$ and $\theta_{n}=0,\vartheta_{n}=0$, it follows that

(26) wn=vn1−w0nγren+11PPγren−1Pγrdn

When $\nu_{n}\geq \frac{\gamma_{re}\left[n\right]\left(1+P\gamma_{rd}\left[n\right]\right)}{\left(\left(1-w_{0}\left[n\right]\right)P\cdot \gamma_{re}\left[n\right]+1\right)\gamma_{rd}\left[n\right]}$ and $\theta_{n}=0,\vartheta_{n}\geq 0$, it follows that $w\left[n\right]=1$.

**Lemma 1.**  *If $\gamma_{sr}\left[n\right]$ is non-increasing, and $\gamma_{rd}\left[n\right]$ is non-decreasing, $\gamma_{re}\left[n\right]$ is non-increasing and non-decreasing over n, for any given dual variables, Alice’s optimal power allocation and UAV’s optimal power allocation factor is as follows:*

(27) $$\begin{array}{c} p_{s}^{*}\left[n\right]={\left[\frac{\beta_{n}}{\eta \ln2}-\frac{1}{\gamma_{sr}\left[n\right]}\right]}^{+},\forall n, \end{array}$$

(28) w*n=vn1−w0nγren+11PPγren−1Pγrdn,∀n,

where η is the parameter that ensures $\frac{1}{N}\sum_{i=1}^{N-1}p_{s}\left[n\right]={\bar{P}}_{s}$, ${\left[\cdot \right]}^{+}=\max\left\{\cdot ,0\right\}$, $\sum_{n=1}^{N-1}p_{s}^{*}\left[n\right]=E_{s}$ and $0\leq w^{*}\left[n\right]\leq 1$.

**Proof of Lemma 1.**  The proof of Lemma 1 can refer to the Lagrange dual function process in 3.3. (1).    □

The proposed algorithm-based gradient descent to solve (27) is summarized in Algorithm 1.

**Algorithm 1** An iterative algorithm based on gradient descent to solve $p_{s}^{*}\left[n\right]$ with fixed $\lambda_{n}$**initialization:**
1. Set Alice’s average output power ${\bar{P}}_{s}$, initial variable η.
2. Set initial dual variables $\lambda_{n}$ to satisfy constraints $\lambda_{n}\geq 0,\sum_{n=2}^{N}\lambda_{n}\leq 1,\forall n$.
3. Set error tolerance ε, the iteration index i=0, maximum number of iterations $i_{\max}$.
**while**
$\;i<i_{\max}\;$
**do**
1. Compute $p_{s}\left[n\right]$ and the gradient $\nabla_{\eta }p_{s}\left[n\right]$ based on (27);
2. Update $\eta_{i+1}$ using the Newton’s iterative method;
3. **If**
$\;\left|\eta_{i+1}-\eta \right|<\varepsilon$
4.      **break**;
5. **else**
6.       i=i+1;
7. **end if**
**end while**
**Output:** η and $p_{s}\left[n\right]$.


Where the convergence tolerance $\varepsilon =10^{-5}$, based on the following rationale:

1. Engineering precision: This tolerance ensures that the computational error in power allocation remains below 0.1%, which significantly exceeds the implementation precision of actual RF chains;
2. Computational efficiency: Sensitivity analysis indicates that when $\varepsilon <10^{-5}$, further improvement in the system secrecy capacity becomes saturated (less than 0.1%), while computational cost increases significantly. Therefore, $10^{-5}$ represents an ideal trade-off point.
3. Coordinated global convergence: To ensure the stability of the outer-layer alternating iteration optimization (with a threshold of $\delta =10^{-4}$), the inner-layer Newton’s method must maintain higher precision, adhering to the principle that ε<δ.

We set $\lambda_{n}=1/\left(N-1\right)$, this setting satisfies the constraint $\lambda_{n}\geq 0,\sum_{n=2}^{N}\lambda_{n}\leq 1,\forall n$, and assigns equal initial weight to the constraint of each time slot.

(2) For the given $p_{s}^{*}\left[n\right]$ and $w^{*}\left[n\right]$, assuming that the Lagrange dual function has a subgradient of $s={\left[s_{2},\cdots ,s_{N}\right]}^{T}$ at ${\left\{\lambda_{n}\right\}}_{n=2}^{N}$, where $s_{n}=\sum_{i=1}^{n-1}\log_{2}\left(1+p_{s}^{*}\left[i\right]\gamma_{sr}\left[i\right]\right)-\sum_{i=2}^{n}R_{d}^{*}\left[i\right],n=2,\cdots ,N$, the optimization problem of the ${\left\{\lambda_{n}\right\}}_{n=2}^{N}$ can be expressed as follows:

(29) $$\begin{array}{cc} \underset{\lambda_{n}}{\min} & \sum_{n=2}^{N}\lambda_{n}s\\ s.t. & \sum_{n=2}^{N}\lambda_{n}\leq 1,\\ \; & \lambda_{n}\geq 0,n=2,\cdots ,N. \end{array}$$

**Lemma 2.**  If $\gamma_{sr}\left[n\right]$ is non-increasing, $\gamma_{rd}\left[n\right]$ is non-decreasing, and $\gamma_{re}\left[n\right]$ is non-increasing and non-decreasing over n, the dual optimal solution ${\left\{\lambda_{n}\right\}}_{n=2}^{N}$ must satisfy $\lambda_{n}^{*}=0,\forall n=2,\cdots ,N-1.$

**Proof of Lemma 2.**  Please refer to the Appendix A.    □

For any $0\leq {\tilde{E}}_{s}\leq E_{s}$, define the total transmission rate of Alice is $R_{s}^{*}\left[n\right]=\sum_{n=1}^{N-1}{\left[\log_{2}\left(\frac{\gamma_{sr}\left[n\right]}{\eta \ln2}\right)\right]}^{+}$ with total transmission power ${\tilde{E}}_{s}$, and $p_{s,n}^{\mathrm{cwf}}\left({\tilde{E}}_{s}\right)\overset{\Delta }{\;=}{\left[\frac{1}{\eta \ln2}-\frac{1}{\gamma_{sr}\left[n\right]}\right]}^{+}$ as the corresponding power allocation for slot *n*, with η satisfying $\sum_{n=1}^{N-1}{\left[\frac{1}{\eta \ln2}-\frac{1}{\gamma_{sr}\left[n\right]}\right]}^{+}={\tilde{E}}_{s}$. This leads to the following result.

**Theorem 1.**  *If $\gamma_{sr}\left[n\right]$ is non-increasing, $\gamma_{rd}\left[n\right]$ is non-decreasing, and $\gamma_{re}\left[n\right]$ is non-increasing and non-decreasing over n, the optimal power allocation can be relaxed without loss of optimality.*

(30) $$p_{s}^{*}\left[n\right]=p_{s,n}^{\mathrm{cwf}}\left({\tilde{E}}_{s}\right)$$

(31) w*n=1−w0nγren+11PPγren−1Pγrdn,∀n,

where ${\tilde{E}}_{s}$ satisfying $R_{s}^{\mathrm{cwf}}\left({\tilde{E}}_{s}\right)=\sum_{n=2}^{N}R_{d}^{*}\left[n\right]$ and $0\leq w^{*}\left[n\right]\leq 1$.

**Proof of Theorem 1.**  Please refer to the Appendix B.    □

The (29) is a typical optimization problem, which can be solved by the interior point method. The proposed algorithm-based alternative and iterative algorithm to solve (29) is summarized in Algorithm 2.

**Algorithm 2** An alternative and iterative algorithm to solve $w\left[n\right]$ and ${\left\{\lambda_{n}\right\}}_{n=2}^{N}$**initialization:**
1. Set initial feasible point ${\left\{w_{0}\left[n\right]\right\}}_{n=2}^{N}$.
2. 
Set UAV’s transmit power *P* and error tolerance δ.
**repeat**
1. Compute $p_{s}\left[n\right]$ by Algorithm 1 and $w\left[n\right]$ based on (28);
2. Update $w_{0}\left[n\right]=w\left[n\right]$;
3. Compute the subgradient s;
4. Update $\lambda_{n}$ using interior point method based on (29)
**until** $\lambda_{n}$ converges to δ.
**Output:** $\left\{\lambda_{n}^{*}\right\}$, $p_{s}^{*}\left[n\right]$ and $w^{*}\left[n\right]$.


Where $w_{0}\left[n\right]=0.5,\forall n.$ This setting assumes that the signal and AN power are equally allocated at the initial moment, serving as an unbiased initialization strategy.

To evaluate the algorithm’s sensitivity to initial values, we conducted Monte Carlo simulations by randomly generating 100 distinct sets of initial points ${\left\{w_{0}\left[n\right]\right\}}_{n=2}^{N}$ and ${\left\{\lambda_{n}\right\}}_{n=2}^{N}$, All experiments converged to the same final solution (with a secrecy capacity difference of less than 0.1%), demonstrating that the algorithm is insensitive to the initial point and exhibits favorable stability.

The Algorithm 2 adopts the relative L2-norm variation of the dual variables as the convergence criterion, where $\delta =10^{-4}$, the selection of the threshold δ is based on the following considerations:

1. A trade-off between numerical accuracy and computational cost: The choice of δ must balance computational accuracy and algorithmic efficiency. An overly small δ (e.g., $10^{-10}$) would lead to unnecessary iterations and increased computation time, while an overly large δ (e.g., $10^{-2}$) may compromise the optimality of the solution;
2. Engineering practice standards: In communication system optimization, when the relative change in the objective function (secrecy capacity) is on the order of $10^{-4}$, its impact on system performance enhancement becomes negligible. According to the convergence properties of first-order optimization methods, the accuracy of the dual variables typically aligns with the accuracy of the objective function. Therefore, we set $\delta =10^{-4}$ to ensure that the algorithm terminates efficiently while achieving sufficient engineering precision.
3. Simulation verification: We tested the convergence behavior for δ ranging from $10^{-2}$ to $10^{-6}$. When $\delta <10^{-4}$, the improvement in secrecy capacity was less than 0.01%, while the number of iterations increased significantly. Hence, δ = $10^{-4}$ represents an ideal compromise.

After obtaining the dual optimal solution ${\left\{\lambda_{n}^{*}\right\}}_{n=2}^{N}$, the throughput of Alice, Bob, and Eve can be obtained through $p_{s}^{*}\left[n\right]$ and $w^{*}\left[n\right]$. We will analyze the following four cases.

*Case 1*: $\beta_{1}^{*}>0$ and $\nu_{N}^{*}>0$, which is equivalent to $\sum_{n=2}^{N}\lambda_{n}^{*}>0$ and $\lambda_{N}^{*}<1$. In this case, both $\left\{\beta_{n}^{*}\right\}$ and $\left\{\nu_{n}^{*}\right\}$ strictly positive vector, (14) and (15) are strictly convex optimization problems, and there exists a unique solution. Furthermore, (14) and (15) demonstrate that the optimal allocation results all satisfy the WF solution.

*Case 2*: $\beta_{1}^{*}=0$ and $\nu_{N}^{*}>0$, which is equivalent to $\lambda_{n}^{*}=0,\forall n$. We can obtain $\beta_{n}^{*}=0$ and $\nu_{n}^{*}=1,\forall n$. In this case, Alice’s optimal power allocation is not unique. Bob’s optimal power allocation factor is given by w*n=1−w0nγren+11PPγren−1Pγrdn,∀n, where $w^{*}\left[n\right]$ must satisfy $0\leq w^{*}\left[n\right]\leq 1$. Under the given power allocation factor, we can obtain Alice’s primal optimal solution that satisfies the information causality constraint.

*Case 3*: $\beta_{1}^{*}>0$ and $\nu_{N}^{*}=0$, which is equivalent to $\lambda_{n}^{*}=0,n=2,\cdots N-1,$ and $\lambda_{N}^{*}=1$. We can obtain $\beta_{n}^{*}=1$ and $\nu_{n}^{*}=0,\forall n$. In this case, Alice’s optimal power allocation solution is classic WF power allocation with a constant water level, i.e., $p_{s}^{*}\left[n\right]={\left[\frac{1}{\eta \ln2}-\frac{1}{\gamma_{sr}\left[n\right]}\right]}^{+},\forall n$, with η satisfying $\frac{1}{N}\sum_{i=1}^{N-1}p_{s}\left[n\right]={\bar{P}}_{s}$. Due to $\nu_{N}^{*}=0$, the optimal solution for Bob will not be unique. We can obtain Bob’s primal optimal solution with Alice’s given power allocation.

*Case 4*: $\beta_{1}^{*}=0$ and $\nu_{N}^{*}=0$ mean that $\lambda_{n}^{*}$ needs to satisfy both $\lambda_{n}^{*}=0,\forall n$ and $\lambda_{N}^{*}=1$ at the same time, which are contradictory to each other. This case is obviously impossible to happen.

### 3.4. Computational Complexity

While the computation of the proposed method in this paper mainly consists of the following parts:

1. Newton iteration for η: O(N).
2. Compute $p_{s}^{*}\left[n\right]$ and $w^{*}\left[n\right]$: O(N).
3. Compute the subgradient s: $O\left(N^{2}\right)$.
4. Interior-Point Method (IPM) for (29): $O\left(N^{3}\right)$.

The overall computational complexity is $O\left(KN^{3}\right)$, Simulation results indicate that, under the set convergence tolerance, the algorithm converges in an average of only K<8 iterations, compared to the traditional SDP method, which typically has a complexity of at least $O\left(N^{6}\right)$, the proposed algorithm exhibits lower computational complexity.

## 4. Simulation and Discussion

In this section, to evaluate the effectiveness of the proposed optimization algorithm, conduct a simulation analysis. The simulation settings parameters are as follows: E=1500m, D=2000m, T=100s, H=100m, ${\bar{p}}_{s}=10\;\mathrm{dBm}$, P=10dBm, the acceleration of the UAV is $a=5\;m/s^{2}$, the maximum speed of the UAV is $V_{\max}=50\;m/s$, the UAV flies from Alice to Bob at a constant altitude *H*. The communication bandwidth per link is 20 MHz with the carrier frequency at 5 GHz, the noise power spectrum density is −169 dBm/Hz and $\gamma_{0}=80\;\mathrm{dB}$. All numerical results are calculated based on the raw simulation data.

Two algorithms were considered as baseline algorithms in the simulation: the throughput maximization algorithm without considering the influence of eavesdroppers, abbreviated as “baseline 1”, which considers maximizing the information reception rate of legitimate destinations as much as possible [20]; The algorithm for maximizing security capacity considering the influence of eavesdropping location, abbreviated as “baseline 2”, which is based on the differences between legitimate channel and eavesdropping channel to maximum security capacity [21].

To quantitatively evaluate the security performance of the algorithm, this paper defines the following core performance metrics. Among them, the eavesdropping capacity suppression percentage is a key indicator for assessing the algorithm’s ability to degrade Eve’s capability, and its calculation method is described below.

Let $R_{e}^{\left(\mathrm{algo}\right)}\left[n\right]$ denote the instantaneous achievable rate of Eve in the *n* time slot under a given algorithm. The cumulative amount of eavesdropped information under that algorithm, denoted as $C_{e}^{\left(\mathrm{algo}\right)}$, is obtained by integrating the instantaneous rate over the communication duration. Since the time is discretized into *N* time slots, the integration translates to

(32) $$C_{e}^{\left(\mathrm{algo}\right)}=\sum_{n=1}^{N}R_{e}^{\left(\mathrm{algo}\right)}\left[n\right]\cdot \Delta t,$$

where Δt=TTNN is the time slot duration.

Consequently, the percentage reduction $\eta_{\sup}$ in eavesdropping capacity achieved by the proposed algorithm compared to the baseline algorithm is as follows:

(33) $$\eta_{\sup}=\left(1-\frac{C_{e}^{\left(\mathrm{proposed}\right)}}{C_{e}^{\left(\mathrm{baseline}\right)}}\right)\times 100\%,$$

This metric reflects the extent to which the proposed algorithm suppresses the total amount of information that can be acquired by the eavesdropper. The closer $\eta_{\sup}$ is to 100%, the more significant the security enhancement achieved.

**Figure 3:**
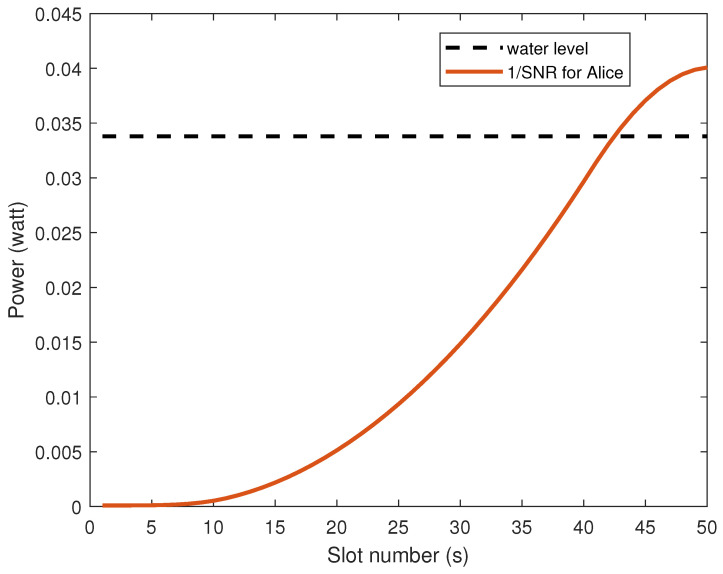
Alice’s optimal power allocation diagram.

Figure 3 shows the WF situation of the optimal power of Alice’s transmitter as the UAV flight time (s) changes. As the UAV moves away from Alice, the Alice–UAV link is affected by the increase in link distance, and the signal-to-noise ratio of the link gradually decreases. When the 1/SNR is higher than the water level, the preset information transmission conditions are no longer met, and power is no longer injected.

**Figure 4:**
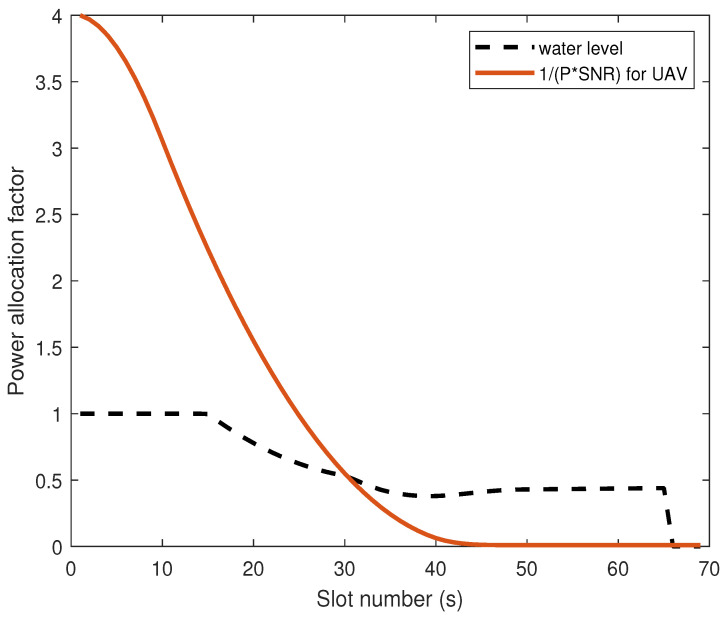
Bob’s optimal power allocation diagram.

**Figure 5:**
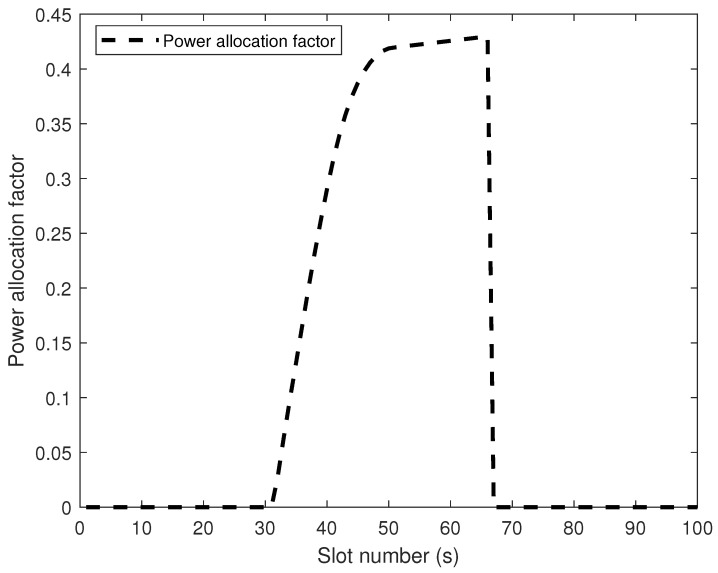
The optimal power allocation factor result chart for the UAV.

**Figure 6:**
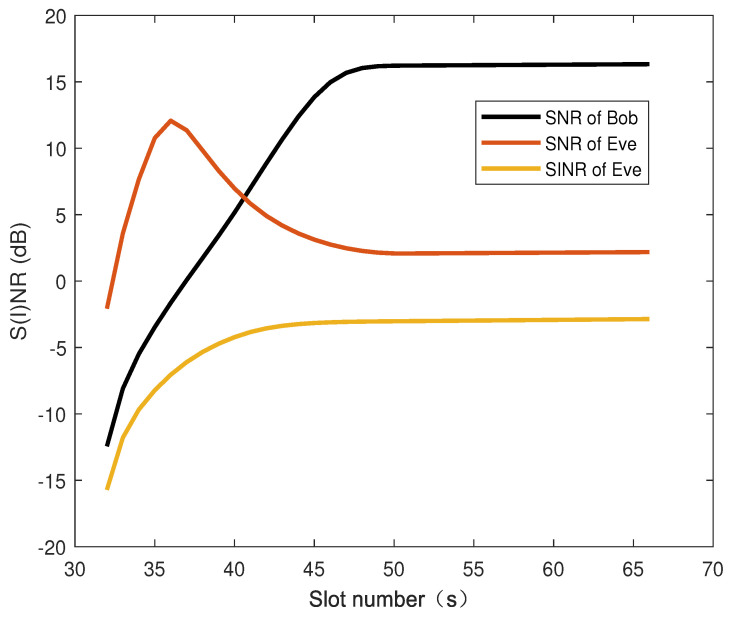
The effect of artificial noise on channel conditions.

Figure 4 illustrates the WF situation of power allocation factor as a function of UAV flight time (s). 32∼40 s, the noise power required to jam Eve increases as the UAV–Eve link distance decreases, resulting in a lower water level. Conversely, 40∼50 s, when the UAV–Eve link distance grows and the UAV–Bob link distance shortens, the noise power diminishes while the information transmission power rises, resulting in a recovery of the water level. 50∼67 s, the UAV arrived and hovered above Bob, but due to the presence of Eve, the UAV still needs to inject AN to suppress the eavesdropping rate. It is noteworthy that during 50∼67 s, the optimal solution for the power allocation factor may exhibit minor fluctuations as the employed algorithm iteratively approaches the global optimum. The optimal power allocation factor result is shown in Figure 5:

Figure 6 illustrates the suppression effect of AN on Eve channel conditions. The proposed algorithm dynamically suppresses eavesdropping channel conditions by controlling the AN injection power, thereby widening the gap between legitimate and eavesdropping channels.

**Figure 7:**
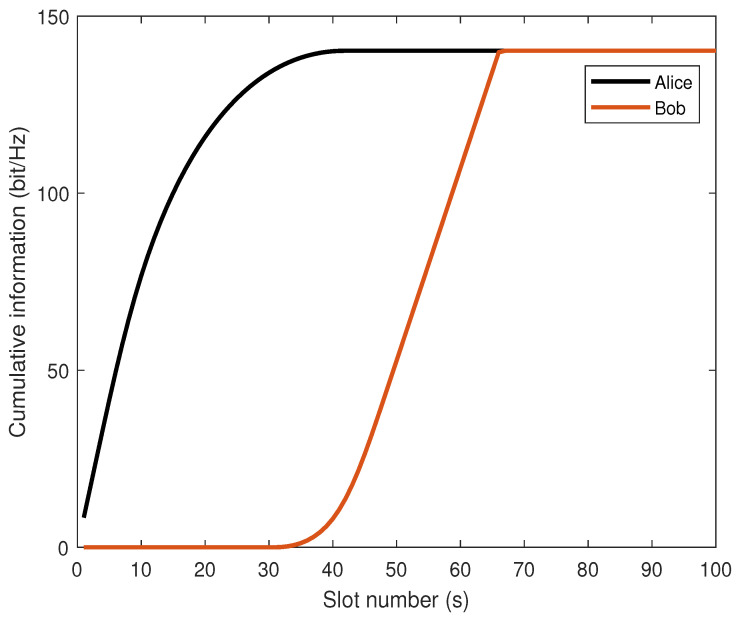
Comparison of cumulative information received/sent.

Figure 7 illustrates the relationship between the cumulative information sent by Alice and the cumulative information received by Bob over time. The total information received by Bob is always less than or equal to the total information sent by Alice, proving that the proposed algorithm satisfies the information causality constraint.

**Figure 8:**
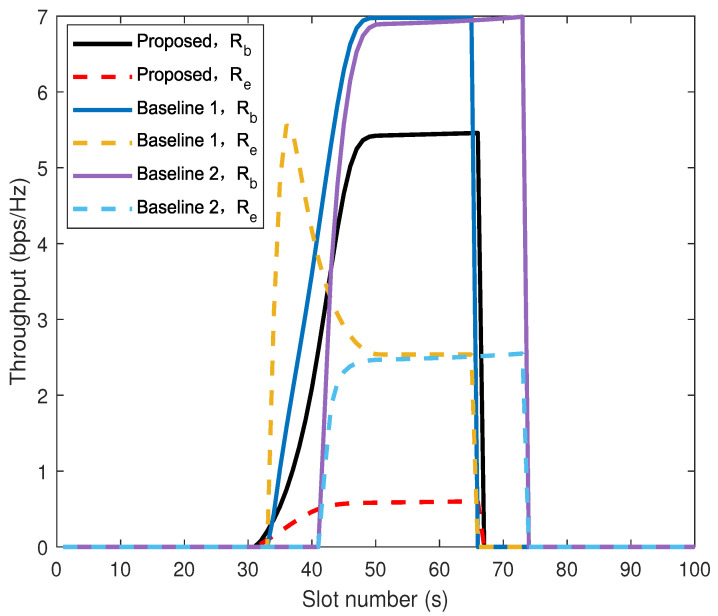
Comparison of throughput between the proposed algorithm and baseline algorithms on Bob and Eve.

Figure 8 shows the comparison of throughput between the proposed algorithm and baseline 1 and 2 for Bob and Eve. The curve of Baseline 1 exhibits an anomalous peak when the UAV flies near Eve. This phenomenon stems from a design flaw in the algorithm: its sole objective is to maximize the amount of information received by Bob, while security risks are not taken into account. When the UAV is closest to Eve, the eavesdropper’s channel quality is optimal. Although the power allocated to this segment is intended to increase Bob’s rate, it simultaneously causes Eve’s interception rate to spike sharply. Given the same total power, due to the UAV using a portion of its power to inject AN, Bob’s information reception rate will be lower than Bob’s information reception rate in baseline 1 and 2. However, minimizing the eavesdropping rate of Eve is equally important. The proposed algorithm achieves a significantly lower eavesdropping rate compared to baseline 2, according to Equation (33), the the calculation shows that the eavesdropping capacity is reduced by 79%. (The cumulative secrecy capacity of the proposed algorithm differs by 7% compared to that of baseline 2.) It is worth noting that the legitimate and eavesdropping channel conditions of baseline 1 are only determined by the link distance. The proposed algorithm introduces AN, expands the scope of secure communication, and improves 9% transmission efficiency. Overall, Algorithm 1 outperforms the two baseline algorithms.

**Figure 9:**
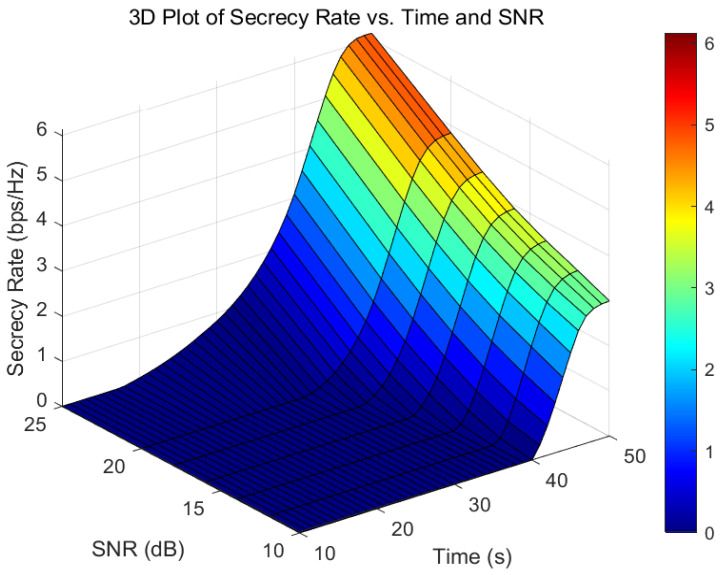
Three-dimensional comparison chart of Secrecy Rate, Time, and SNR.

Figure 9 reveals the impact of temporal characteristics and signal-to-noise ratio (SNR) on the system’s secure transmission performance. Experimental results demonstrate that for every 2 dB increase in the total system SNR: (1) the average secure rate improves by approximately 0.37 bps/Hz; (2) the UAV’s average secure communication range (the interval during which the channel conditions of Bob are superior to those of Eve) expands by 35%; (3) the system’s communication efficiency enhances by 4.5%.

**Figure 10:**
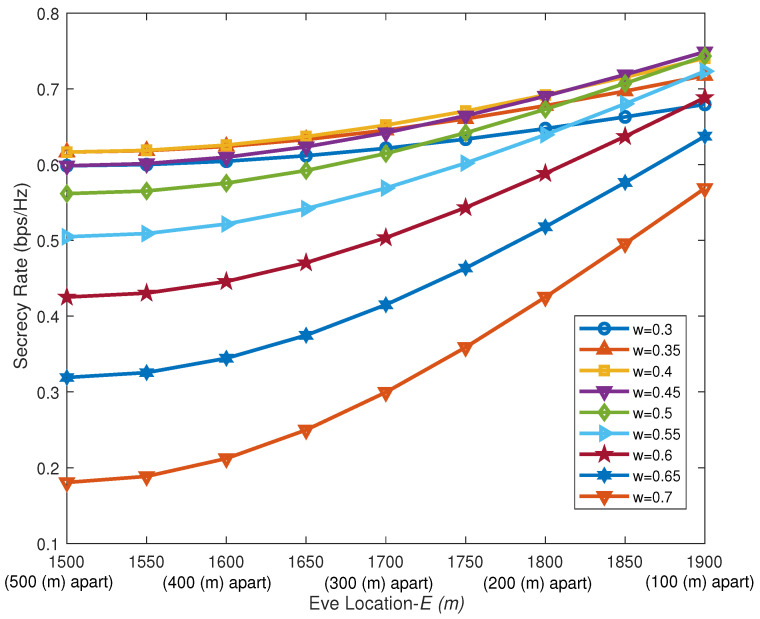
Impact of different Eve positions on algorithm performance.

Figure 10 illustrates the impact of different power allocation factors on the system secrecy rate as the distance between Eve and Bob decreases (i.e., the value of E increases). Assuming the UAV hovers at $\left(1500,0,100\right)$ and the total power is constant, Eve’s channel condition is superior to Bob’s initially. As Eve gradually moves closer to Bob, the interference effect of AN on Eve weakens. However, the increasing distance of the UAV–Eve link also leads to a degradation of Eve’s channel quality.   In summary, the system’s secrecy rate is enhanced. As the power allocation factor decreases, the proportion of power allocated to AN increases. This actively amplifies interference to artificially widen the disparity between Bob’s channel and Eve’s channel, thereby compensating to some extent for the security performance loss caused by Eve’s proximity. However, as the proportion of AN power continues to increase, the marginal improvement in secrecy rate it can provide gradually diminishes. The secrecy capacity curves corresponding to different power allocation factors may intersect. This occurs because the suppression effect of a high-power AN on the Eve can no longer outweigh the enhancement in the upper limit of transmission rate brought by allocating more power to the information-bearing signal. Consequently, under a total power constraint, a trade-off between the power allocated to the information signal and that allocated to the AN is necessary. This balance aims to maximize the secrecy capacity while simultaneously improving the information transmission efficiency.

## 5. Conclusions

This paper proposes an AN-assisted UAV relay secure communication system, which effectively enhances physical-layer security by injecting AN through the UAV during signal forwarding. Leveraging real-time CSI and dynamic UAV positioning, the system adaptively adjusts the power allocation factor to precisely control interference on the eavesdropping channel, effectively addressing the challenge of suppressing the eavesdropping rate when the eavesdropper is close to the legitimate destination. To tackle the non-convex optimization problem induced by information causality constraints and Shannon capacity, a novel algorithm integrating relaxation variables and SCA is designed, successfully deriving an Algorithm for optimal power allocation with AN-assistance. Simulation results demonstrate that, under the same total power constraint, the proposed scheme significantly reduces the eavesdropping channel capacity, expands the secure communication range, and simultaneously improves the system’s security performance and communication efficiency, providing a new theoretical and practical foundation for optimizing the security of UAV relay communication systems. In the future, deterministic optimization and stochastic covert communication strategies can be integrated to construct a multi-dimensional defense-in-depth architecture.

## Data Availability

The original contributions presented in this study are included in the article. Further inquiries can be directed to the corresponding author(s).

## Appendix A

We use the method of contradiction to prove Lemma 2. Assuming that 2≤i≤N−1, $\lambda_{i}^{*}>0$. Thus, the transmission rate of Alice and Bob can be expressed as

(A1) $$R_{s}^{*}\left[n\right]={\left[\log_{2}\left(\frac{\beta_{n}^{*}\gamma_{sr}\left[n\right]}{\eta \ln2}\right)\right]}^{+},n=2,\cdots ,N$$

(A2) $$R_{d}^{*}\left[n\right]=\log_{2}\left(\frac{\tau_{n}\gamma_{rd}\left[n\right]}{\gamma_{re}\left[n\right]}\right),n=2,\cdots ,N$$

where $\tau_{n}=v_{n}\left(P\cdot \left(1-w_{0}\left[n\right]\right)\gamma_{re}\left[n\right]+1\right)$

Since both $\beta_{n}^{*}$ and $\gamma_{sr}\left[n\right]$ is non-increasing over *n*, the $R_{s}^{*}\left[n\right]$ is non-increasing:

(A3) $$\sum_{n=1}^{N-1}R_{s}^{*}\left[n\right]\geq \left(N-1\right)R_{s}^{*}\left[N-1\right].$$

Under the above assumption, the information causality constraints are active from *n* to N−1, UAV must immediately forward all received data in each time slot:

(A4) $$\sum_{n=1}^{i-1}R_{s}^{*}\left[n\right]=\sum_{n=2}^{i}R_{d}^{*}\left[n\right].$$

This implies that the UAV cannot store data, which limits the flexibility of $w\left[n\right]$.

**Lemma A1.** 
*If $\gamma_{sr}\left[n\right]$ is non-increasing, $\gamma_{rd}\left[n\right]$ is non-decreasing, and $\gamma_{re}\left[n\right]$ is non-increasing and non-decreasing over n, before the UAV completes the information transmission task, $R_{d}^{*}\left[n\right]$ is non-decreasing.*

**Proof of Lemma A1.** Assuming the closest slot between UAV and Eve is $n_{1}$, $\gamma_{re}\left[n\right]$ is increasing within $2\leq n\leq n_{1}$ and non-increasing within $n_{1}\leq n\leq N-1$, and $\gamma_{rd}\left[n\right]$ is non-decreasing over *n*. (1) If $\gamma_{re}\left[n_{2}\right]<\gamma_{re}\left[n_{3}\right]$, i.e., $2\leq n_{2}\leq n_{3}\leq n_{1}$, we need to increase AN to suppress higher eavesdropping gain, so there are $w\left[n_{2}\right]>w\left[n_{3}\right]$. Due to the limited power *P*, even if $w\left[n_{2}\right]=0$, $SINR_{re}\left[n_{2}\right]$ may still be greater than $\gamma_{rd}\left[n_{2}\right]$. During this time period, the secrecy capacity is negative, which is not allowed for maximizing the secrecy capacity, $R_{d}^{*}\left[n\right]=0,2\leq n\leq n_{2}.$ Assuming the existence of $n_{2}<n_{3}<n_{4}<n_{1}$ makes $R_{d}^{*}\left[n_{3}\right]>R_{d}^{*}\left[n_{4}\right]>0$. During this time period, the eavesdropping channel can be suppressed by rationally allocating power *P* to AN, thereby achieving $SINR_{re}\left[n_{3}\right]<\gamma_{rd}\left[n_{3}\right]$, while ensuring a positive secrecy capacity. If $\gamma_{re}\left[n_{3}\right]<\gamma_{re}\left[n_{4}\right]$, $\gamma_{rd}\left[n_{3}\right]<\gamma_{rd}\left[n_{4}\right]$, for the same $w\left[n\right]$, we have $SINR_{re}\left[n_{3}\right]<SINR_{re}\left[n_{4}\right]$. Therefore, we can increase the power used for information transmission $w\left[n_{3}\right]<w\left[n_{4}\right]$. There will be $R_{d}^{*}\left[n_{3}\right]<R_{d}^{*}\left[n_{4}\right]$, which contradicts our hypothesis. (2) Assuming the existence of $n_{1}<n_{2}<n_{3}<N-1$ makes $R_{d}^{*}\left[n_{2}\right]>R_{d}^{*}\left[n_{3}\right]>0$. If $\gamma_{re}\left[n_{2}\right]\geq \gamma_{re}\left[n_{3}\right]$, $\gamma_{rd}\left[n_{2}\right]\leq \gamma_{rd}\left[n_{3}\right]$, the channel quality for Eve degrades, we can increase the power used for information transmission, so there are $w_{0}\left[n_{2}\right]\leq w_{0}\left[n_{3}\right]$. There will be $R_{d}^{*}\left[n_{2}\right]\leq R_{d}^{*}\left[n_{3}\right]$, which also contradicts our hypothesis. In summary, the $R_{d}^{*}\left[n\right]$ is non-decreasing.

(A5) $$\sum_{n=2}^{N}R_{d}^{*}\left[n\right]\leq \left(N-1\right)R_{d}^{*}\left[N\right]$$

From Equations (A3) and (A5), we can obtain $\sum_{n=2}^{N}R_{d}^{*}\left[n\right]>\sum_{n=1}^{N-1}R_{s}^{*}\left[n\right]$, which clearly violates the information causality constraint. Therefore, the value of $\lambda_{n}^{*}$ satisfies $\lambda_{n}^{*}=0,\forall n=2,\cdots ,N-1$. This completes the proof of Lemma 2. □

## Appendix B

For Case 3, Alice’s optimal power allocation $p_{s}^{*}\left[n\right]$ is given by the classic WF solution with all power budget, i.e., $p_{s}^{*}\left[n\right]=p_{s,n}^{\mathrm{cwf}}\left(E_{s}\right),\forall n.$ The transmission rate of Alice is $R_{s}^{*}\left[n\right]={\left[\log_{2}\left(\frac{\gamma_{sr}\left[n\right]}{\eta \ln2}\right)\right]}^{+},n=1,\cdots ,N-1$, with η denoting the water level. The optimal power of the UAV can be obtained by $R_{s}^{*}\left[n\right]$.

(A6) maxpsnn=1N−1,wnn=1N−1∑n=2NRdn−∑n=2NRew0n−∑n=2Nγrenwn−w0n1−w0nγren+11PPln2s.t.∑i=2nRdn≤∑i=1n−1Rs*n,n=2,⋯,N,Rdn≤Rdwn,n=2,⋯,N,0≤wn≤1,n=2,⋯,N,

According to Lemma 2, the information causality constraint can be ignored from slot 2 to slot N−1.

(A7) maxpsnn=1N−1,wnn=1N−1∑n=2NRdn−∑n=2NRew0n−∑n=2Nγrenwn−w0n1−w0nγren+11PPln2s.t.∑n=2NRdn≤∑n=1N−1Rs*n,Rdn≤Rdwn,n=2,⋯,N,0≤wn≤1,n=2,⋯,N,

**Lemma A2.** 
*If $\gamma_{sr}\left[n\right]$ is non-increasing, $\gamma_{rd}\left[n\right]$ is non-decreasing, and $\gamma_{re}\left[n\right]$ is non-increasing and non-decreasing over n, before the UAV completes the information transmission task, problem (A6) and (A7) is equivalent.*

**Proof of Lemma A2.** Note that problem (A7) is a relaxation of (A6). If the optimal solution of (A7) is also applicable to (A6), then problem (A7) and (A6) are equivalent. Suppose, on the contrary, assume the information causality constraint is active on partial time slots from 2 to N−1. Suppose there exists $n_{1}$ such that both $\sum_{i=1}^{n_{1}-1}R_{s}^{*}\left[i\right]<\sum_{i=2}^{n_{1}}R_{d}^{*}\left[i\right]$ and $\sum_{i=2}^{n_{1}-1}R_{d}^{*}\left[i\right]\leq \sum_{i=1}^{n_{1}-2}R_{s}^{*}\left[i\right]$. Then we have $R_{d}^{*}\left[n_{1}\right]>R_{s}^{*}\left[n_{1}-1\right]$. Appendix A has proven that $R_{s}^{*}\left[n\right]$ is non-increasing and $R_{d}^{*}\left[n\right]$ is non-decreasing, we have

(A8) $$\sum_{i=n_{1}}^{N-1}R_{s}^{*}\left[i\right]<\sum_{i=n_{1}+1}^{N}R_{d}^{*}\left[i\right]$$

Combining the assumption $\sum_{i=1}^{n_{1}-1}R_{s}^{*}\left[i\right]<\sum_{i=2}^{n_{1}}R_{d}^{*}\left[i\right]$, we have

(A9) $$\sum_{i=1}^{N-1}R_{s}^{*}\left[i\right]<\sum_{i=2}^{N}R_{d}^{*}\left[i\right],$$

which contradicts the information causality constraint of problem (A6). Thus, the assumption is invalid. This completes the proof of Lemma A2. □

We can obtain the problem (A7) and (A6) are equivalent, the optimal solution of (A7) is also the optimal solution to (A6). This completes the proof of Theorem 1.
